# Possible association of the *TERT* promoter polymorphisms rs2735940, rs7712562 and rs2853669 with diabetes mellitus in obese elderly Polish population: results from the national PolSenior study

**DOI:** 10.1007/s13353-018-0450-9

**Published:** 2018-06-25

**Authors:** Ewa Gutmajster, Jerzy Chudek, Aleksandra Augusciak-Duma, Malgorzata Szwed, Aleksandra Szybalska, Malgorzata Mossakowska, Monika Puzianowska-Kuznicka, Andrzej Wiecek, Aleksander L. Sieron

**Affiliations:** 1 Department of Molecular Biology and Genetics, School of Medicine in Katowice, Medical University of Silesia in Katowice, 18 Medykow Street, 40-752 Katowice, Poland; 2 Department of Internal Medicine and Oncological Chemotherapy, School of Medicine in Katowice, Medical University of Silesia in Katowice, 40-027 Katowice, Poland; 3 Department of Human Epigenetics, Mossakowski Medical Research Centre, 02-106 Warsaw, Poland; 4 International Institute of Molecular and Cell Biology, 02-109 Warsaw, Poland; 5 Department of Geriatrics and Gerontology, Medical Centre of Postgraduate Education, 01-813 Warsaw, Poland; 6 Department of Nephrology, Transplantation and Internal Medicine, Medical University of Silesia, 40-027 Katowice, Poland

**Keywords:** Diabetes mellitus, Human aging, *hTERT*, Telomerase, Telomeres

## Abstract

One of the markers of aging is lymphocyte telomere length (LTL), which is affected by genetic constitution of the organism and environmental conditions, such as development and diseases, including diabetes. The relationship of the later seems to be bilateral. The enzyme responsible for the maintenance of telomere length is a subunit of telomerase—telomerase reverse transcriptase (TERT). The aims of the present study were to (1) determine the influence of the *TERT* promoter sequence SNP variants on relative telomere length (RTL) in an elderly Polish population and (2) explore the potential associations of the SNPs with the type 2 diabetes mellitus (T2DM) in the obese individuals. Two highly homogenous subgroups of PolSenior participants were investigated, the first constituted 70 relatively healthy respondents and the second 70 individuals with T2DM. Telomere length ratio (T/S value) was measured; 1.5 kb part upstream of the transcription start site of the *TERT* promoter was sequenced, and the frequencies of polymorphisms were calculated and compared against analysed data. Low-frequency SNPs were evaluated but excluded from further comparative analyses to RTL and glucose metabolism markers. No significant difference in telomere length was found between the two studied subgroups. Univariate statistical analyses showed only a weak association of environmental or genetic factors altering this marker of aging. Approximate frequency of four SNPs in *TERT* promoter sequence was assessed in Polish population aged 65–95 years, but three of them (rs2735940, rs7712562 and rs2853669) were selected for further analyses. The SNP selection was based on their minor allele frequencies in general population and on published data. The univariate analysis has revealed that carriers of CC SNP (rs2853669) have had the shortest RTL in the T2DM group. Multivariate analysis has also revealed that the genetic effect of *TERT* promoter CC SNP was strengthened by the incidence of T2DM. The additional variation in RTL in paired groups indicates that in addition to T2DM and genetics, there are other factors contributing to development of the age-related diseases.

**Electronic supplementary material:**

The online version of this article (10.1007/s13353-018-0450-9) contains supplementary material, which is available to authorized users.

## Introduction

The dynamic of telomeres’ length and their age-dependent shortening provide valuable insights into the pathogenesis of chronic inflammatory processes (Zhang et al. 2016) and consequently an aging-related disorders (Ishikawa et al. 2016), such as arterial hypertension (Ma et al. 2015) or cardiovascular disease (Nilsson et al. 2013). Although a growing body of evidence supports an association between short telomeres and type 2 diabetes mellitus (T2DM), most studies have been cross-sectional by nature, trying to answer whether the metabolic disturbances of T2DM cause telomere attrition or if the shorter telomeres lead to higher risk of T2DM. Biological hypotheses address both scenarios. Short telomeres may lead to premature cell senescence, resulting in the reduced cell mass and subsequently impaired insulin secretion and glucose tolerance (Elks and Scott 2014). Conversely, elevated blood glucose concentrations increase oxidative stress and potentially interfere with telomerase function leading to shorter telomeres. On the other hand, high concentration of blood glucose alone, as shown in cultured human fibroblasts, did not cause telomere shortening. However, it was significantly accelerated in cell cultures containing the pro-inflammatory cytokine, interleukin 1 beta (Salpea et al. 2013).

The normalized telomere to centromere signal ratio, determined for adipocytes by the method of in situ hybridization, was reduced in T2DM patients when compared to control ones. It, also, negatively correlated with blood glycated haemoglobin concentration (HbA1c) (Tamura et al. 2016a). Using autopsy samples, telomere length in pancreatic islet cells β and α were found shorter in patients with diabetes mellitus (DM) than in non-diabetic individuals (Tamura et al. 2016b). However, the relation between the relative lymphocyte telomere length (RTL) and the presence of diabetes was questioned in some reports. The results of the study on the US general population lead to the conclusion that RTL was not associated neither with diabetic status, its duration period, medical treatment, nor telomere attrition. Therefore, it was neither a cause nor a consequence of diabetes (Menke et al. 2015).

Although the hypothesis linking obesity and especially coexisting T2DM with accelerated telomere shortening is commonly accepted, the high inter-individual variability of RTL makes it difficult to confirm in a cohort studies. A significant positive association between shorter telomeres and T2DM risk was demonstrated in meta-analysis of 429 records (Willeit et al. 2014) but only in the quartile of participants with the shortest telomeres. In parallel study of a cohort of 2721 elderly subjects, it has been revealed that the shortening of RTL correlated with obesity. However, the correlation was significant with some obesity-related parameters, such as body fat percentage, subcutaneous fat and plasma leptin concentration but not with the others, such as BMI and visceral fat. Therefore, the mutual interplay between obesity and RTL is still uncertain (Njajou et al. 2012; Müezzinler et al. 2014). Only few studies investigating the association between lymphocyte telomere length (LTL) and obesity reported that weight gain, rather than the obese status per se, is the more important factor affecting telomere shortening rates (Buxton et al. 2011). Similarly, in the case of patients with T2DM (501 patients) diagnosed and medically treated, the LTL seemed to be correlated rather with diabetic complications than with the risk of T2DM itself (Testa et al. 2011). The shorter was the LTL baseline; the more pronounced and significant was the insulin resistance over the follow-up period. This effect was additive to that of BMI in parallel twins’ studies (Verhulst et al. 2016). Thus, telomere attrition could provide additive prognostic information on mortality risk in T2DM patients (Bonfigli et al. 2016).

Relatively large variability of RTL could be influenced not only by the above listed factors. The obesity is influenced not only by diet, physical activity, the type of diabetes, etc., but also by sex (Gardener et al. 2014), the type of analysed cells (Svenson et al. 2011; Meyer et al. 2016) and the cell proliferative activity in the analysed tissue. The RTL results depend also on the measurement method (Gutmajster et al. 2013; Rode et al. 2015; Mazidi et al. 2017), the DNA preparation (Raschenberger et al. 2016) and genetic constitution of an organism. RTL is a heritable trait with variability ranging from 34 to 82% and depends on a number of specific genetic variants associated with RTL, including variations in sequence of genes such as *TERT*, *TERC*, *OBFC1*, *CTC1*, *CEP95* and *SMURF2* (Codd et al. 2013; Lee et al. 2013; Zhou et al. 2016). The dynamics of RTL undergo the age-dependent shortening at remarkably rapid rates of attritions until the first 20 years of life. However, inter-individual variation in the initial length of telomeres was remarkable, in spite of its high heritability (Liu 2014). Telomere length was stable in the healthy old (range 61–75 years) and oldest old individuals (range 76–91 years) when compared with the younger ones (Houben et al. 2011; Franzke et al. 2015). There was also less of RTL variation between men and women. Slower telomere attrition rate in women resulted from the oestrogen protective function on the telomere length, which is not the case in post-menopausal women population (Gardener et al. 2014).

SNP analyses, always, are conducted on as big, as possible populations; therefore, in our work to avoid the low power of statistical tests, instead of enlarging the tested population, we screened the entire available to us population for participants that constituted group as homogenous as possible. The human telomerase reverse transcriptase gene sequence (*hTERT*), coding the catalytic subunit of telomerase holoenzyme, was the target in this study. The enzyme has been defined as the rate-limiting factor in regulating telomerase activity in maintaining the telomere length (Ozturk et al. 2017). No previously reported mutations leading to the telomeropathies (Opresko and Shay 2017) were expected. Also, both T2DM patients and controls with cancer histories were excluded from the study (Heidenreich et al. 2014). Additionally, 1100 bps DNA fragment upstream of the ATG start codon of the gene has been well characterized by others (Wick et al. 1999; Lewis and Tollefsbol 2016). The *hTERT* promoter sequence variants were reported as related to premature telomere shortening (Melicher et al. 2015), increased risk of cancer (Heidenreich et al. 2014) and cardiovascular diseases (CAD) (Bressler et al. 2015). Some polymorphisms were described as of no-clinical significance; however, three polymorphic changes (rs2853669, rs3215401, rs2735940) were found to influence telomerase expression (Matsubara et al. 2006a; Helbig et al. 2017). Nevertheless, there is yet no such data available for diabetes. Recently, additional functions of *hTERT*, beyond the maintenance of chromosome stability, have been explored. Telomerase/TERT may act as a transcription modulator through its interaction with transcription factors p65, β-catenin or Brahma-related gene-1 (BRG1) that might regulate transcription of some other genes. TERT is able to form different complexes in different cell contexts and regulate the gene expression in certain pathways (Zhou et al. 2014). TERT has also been shown to shuttle dynamically between different cellular compartments, under increased oxidative stress (Singhapol et al. 2013). Inhibition of *TERT* expression reduced basal 2-deoxyglucose uptake by 50% in human and mouse cell lines, while its overexpression upregulated glucose uptake by 3.25-fold. Therefore, loss of *TERT* expression (e.g. in diabetes or aging) may accompany insulin sensitivity and glucose uptake (Shaheen et al. 2014). It has been also postulated that *TERT* overexpression could induce cell survival and therefore to be applied to ease diabetes mellitus and its vascular complications (Qi Nan et al. 2015).

In this work, we hypothesize that (1) differences in RTL, previously seen by others, between controls and T2DM patients are results of inflammation and oxidative stress caused among others by obesity; therefore, if all participants are obese, no changes in RTL length between both groups should be detected. (2) In clinically homogenous groups, where the effects of genetic constitution of individuals are more pronounced, changes detected in the sequence of the *TERT* promoter affect telomerase activity in both telomeres’ length and glucose transport; thus, increasing the risk of T2DM in elder and obese individuals could serve as its prognostic marker.

## Materials and methods

### Participants

The analyses were carried out on a group of participants carefully selected from the cohort of the PolSenior study. Information on age, sex, socio-demographic characteristics, medical history, health status, family history and lifestyle were obtained based on detail questionnaires in a standardized manner (Bledowski et al. 2011).

In the group of 1842 subjects with assessed telomere length (data not shown), 277 participants were treated for diabetes and among them 140 individuals were obese, according to WHO criteria. The number of obese participants without T2DM was 411. From this cohort, participants with inflammatory conditions, namely rheumatoid diseases, acute and chronic infections, history of cancer, stroke, congestive heart failure, dementia or chronic obstructive pulmonary disease (except hypertension) were excluded. The group of T2DM included only patients previously diagnosed and already pharmacologically treated for diabetes with insulin. The inclusion criterion was the coexistence of insulin resistance (HOMA-IR values above 2.5). Selected subjects from the group without T2DM were matched with age, sex and in equal proportion of men and women.

The final group, strictly fulfilling the presented above criteria, consisted of 140 participants, of which 70 were obese with T2DM and 70 were control obese without T2DM. For this group, the power of statistical tests, for calculated variance, was satisfied. Specifically, 28 of age 65–69 years included 15 with T2DM, 28 of age 70–74 years included 14 with T2DM, 28 of age 75–79 years included 13 with T2DM, 28 of age 80–84 years included 14 with T2DM and 28 of age 85–89 and 90–95 years included 14 with T2DM. Detailed characteristics of both groups are presented in Table 1.

**Table 1:** Characteristics of obese controls and obese diabetic (T2DM) elderly subjects. Data presented as mean ± SD or percentages

Parameter	Controls (*n* = 70)	T2DM subjects (*n* = 70)	*p* value
Questionnaire-based and anthropometric measures			
Age (years)	76.8 ± 7.8	76.9 ± 7.7	NS
Sex			NS
Female	54%	47%	
male	46%	53%	
Body mass index (kg/m^2^)	34.7 ± 3.3	35.9 ± 4.0	NS
Smoking status			NS
Never/ex smoker	87%	89%	
Current smoker	13%	11%	
Systolic blood pressure (mmHg)	145.8 ± 20.6	151.5 ± 22.1	NS
Diastolic blood pressure (mmHg)	87.9 ± 12.4	84.4 ± 8.9	NS
Treated hypertension			
No	83%	87%	NS
Yes	17%	13%	
Fibrate intake	0%	4.3%	NS
Statins intake	30%	37%	NS
Lipid profile			
HDL cholesterol (mg/dl)	47.0 ± 10.9	47.4 ± 12.4	NS
LDL cholesterol (mg/dl)	106.6 ± 41.8	118.8 ± 42.5	0.039
Total cholesterol (mg/dl)	202.5 ± 53.1	184.2 ± 47.6	0.017
Triglycerides (mg/dl)	144.9 ± 70.5	138.9 ± 50.0	NS
Markers of inflammation			
hsCRP (high-sensitivity C-reactive protein) (mg/l)	5.2 ± 5.2	4.7 ± 7.0	NS
IL-6 (pg/ml)	3.2 ± 1.9	3.2 ± 2.6	NS
White blood cells count (106/l)	6.5 ± 1.6	6.7 ± 1.7	NS
Markers of oxidative stress			
Uric acid (mg/dl)	5.7 ± 1.6	5.8 ± 1.5	NS
Markers of glucose metabolism			
Fasting plasma glucose (mg/dl)	95.4 ± 11.7	136.3 ± 44.4	< 0.001
Insulin (μU/ml)	9.2 ± 5.2	29.4 ± 53.9	< 0.05
Adiponectin (μg/ml)	11.1 ± 6.1	9.3 ± 4.6	NS
HOMA-IR (homeostatic model assessment of insulin resistance)	3.9 ± 2.5	7.6 ± 7.2	< 0.001
Markers of cell aging			
Relative telomere length (RTL)	78.1 ± 68.1	99.5 ± 93.7	NS
Log RTL	4.04 ± 0.86	4.36 ± 0.87	NS

*NS* not significant

### DNA isolation

Whole blood samples were obtained from all participants. Genomic DNA was extracted by salting-out method and stored at − 80 °C. The concentration and purity of the DNA were assessed by UV spectroscopy (Nanodrop, Thermo Fisher Scientific Inc., Wilmington, DE, USA).

### The TL assay

In white blood cells, TL was measured using the real-time quantitative polymerase chain reaction (Q-PCR) as described previously (Gutmajster et al. 2013). Briefly, the amount of telomeric DNA (T) was divided by the amount of single-copy control gene DNA (S) which encodes acidic ribosomal phosphoprotein P0 (36B4, accession number NC_000012.12), producing a relative measurement of the telomere length (T/S ratio). Results were related to a control sample, used for standard curve generation. The quality of PCR products was assessed by the melting curve analysis. All samples were run in triplicates, and the control sample (Human Genomic DNA, ROCHE, Germany) was run in each experiment to ensure correct normalization among experiments.

### Promoter genotyping

DNA amplification was performed using the mix OptiTaq™ PCR Master Mix (Eurix®) as follows: 5 min at 94 °C, 35 cycles at 94 °C for 30 s, 56 °C for 30 s, 72 °C for 1 min 30 s, with a final step at 72 °C for 7 min. Primer sequences were as follows: forward primer for *TERT* (TERT_F): 5′ATTCGACCTCTCTCCGCTGG3′; reverse primer (TERT_R): 5′CTGGAAGGTGAAGGGGCAG3′. PCR product was treated with exonuclease/alkalic phosphatase mix (Eurix®).

The sequencing PCRs were performed with the BigDye v.3.1 (Fisher Scientific, USA) with internal primers and sequences thanks to courtesy of Prof. Grzybowska (Varadi et al. 2009). Products were cleaned with the BigDye Terminator X (Fisher Scientific, USA). DNA sequencing was performed using Sanger’s technique and analyzed with the ABIPrism3130xl instrument (Fisher Scientific, USA). Results were analyzed with the Blast software (NCBI, USA).

### Laboratory measurements

Serum total cholesterol, LDL cholesterol, HDL cholesterol, triglycerides, glucose, uric acid and C-reactive protein concentrations were assessed by an automated system (Modular PPE, Roche Diagnostics GmbH, Mannheim, Germany) in a single certified laboratory.

Serum insulin concentration was assessed by electrochemiluminescence method (ECLIA) using commercially available kits and the Cobas E411 analyzer (Roche Diagnostics GmbH, Mannheim, Germany). Homeostatic model assessment of insulin resistance (HOMA-IR) was calculated with the standard formula: HOMA-IR = fasting serum insulin (μIU/ml) × fasting glucose (mg/dl)/405.

The plasma concentration of interleukin 6 (R&D Systems, Mineapolis, MN, USA) and adiponectin (B-Bridge International Inc., San Jose, CA, USA) was measured by the immunoenzymatic (ELISA) method.

### Data analysis

Results from both the assays and tests and from the questionnaire-derived data on gender, age, body mass index (BMI), blood pressure and smoking status were analysed. Characteristics of the study population are presented as mean and standard deviation (SD) or median and lower-upper quartile (for non-normal distribution of the data). Categorical variables are reported as frequency and percentage. Pearson’s or Spearman’s correlation coefficients were calculated whenever necessary. Comparison between mean values in the two groups (patients and controls) according to RTL was evaluated using the Student’s *t* test or the Mann-Whitney *U* test when applicable. The association between *TERT* genotypes and variables of interest was initially assessed using multivariate logistic regression analysis, and then comparisons of groups were performed using chi-square test. The lowest analysed minor allele frequency (MAF) was accepted as 0.23, and the calculated power of the test was 0.81, which is the value statistically acceptable for the number of participants in this study (140). Univariate analysis of the association of RTL, anthropometric and biochemical findings between *TERT* genotypes of SNPs was conducted using Pearson correlation. For each SNP, Hardy-Weinberg equilibrium was assessed according to methods described elsewhere www.oege.org/software (Rodriguez et al. 2009). The association between *TERT* genotypes and T2DM risk was determined by calculating the odds ratio (OR) and 95% confidence interval (CI) using binary logistic regression. All the statistical analyses were performed using Statistica v.12 (StatSoft, DELL, USA), and the criterion for statistical significance was *p* < 0.05.

## Results

### Characteristics of the study groups

RTL level was higher in men (99.9 ± 92.4, log RTL 4.35 ± 0.84) than in women (77.9 ± 70.1, log RTL 4.06 ± 0.89), with relatively high variance and the *p* values not significant. Also, no statistically significant correlation between RTL and age was found as expected (Table 1).

T2DM and control groups differed significantly only in respect of the markers of glucose metabolism, such as fasting plasma glucose concentration, HOMA-IR (each *p* < 0.001), serum insulin (*p* < 0.05), total cholesterol and LDL cholesterol (*p* < 0.05) concentrations (Table 1).

RTL value was higher in T2DM patients group (99.5 ± 93.7, log RTL 4.36 ± 0.87) than in control group (78.1 ± 68.1, log RTL 4.04 ± 0.86) but without statistical significance (Table 1).

### Distribution of genotypes within study subjects

Seven already described polymorphisms within the analysed *TERT* promoter fragment were detected: rs2735940, rs3215401, rs7712562, rs33958877, rs35161420, rs35226131 and rs2853669. Four of them, rs3215401, rs33958877, rs35161420 and rs35226131, were excluded from further analyses due to their low MAFs (Table S1).

The rs2735940 polymorphism showed distribution against the Hardy-Weinberg law in both the diabetic participants and in the control group. Additionally, distribution of MAF in the group of healthy control subjects differed significantly from those in general European populations (Table S1).

### Biochemical characteristics according to genotypes

**Fig. 1:**
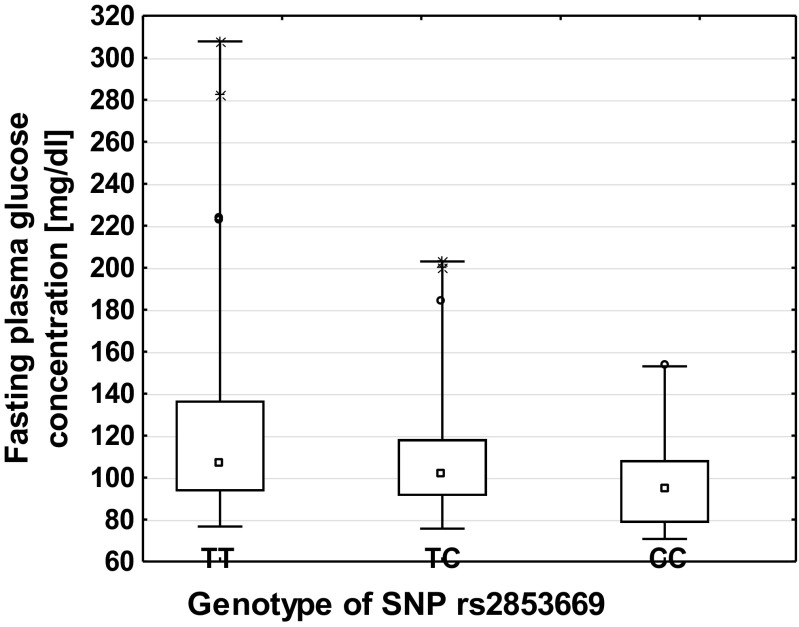
Correlation of fasting plasma glucose concentration with the genotypes of the rs2853669 polymorphism in combined study group. The values are median. The outer boxes mark 25–75%. Circles indicate Det. X that indicates Ext. Min and max values are marked by error bars

**Fig. 2:**
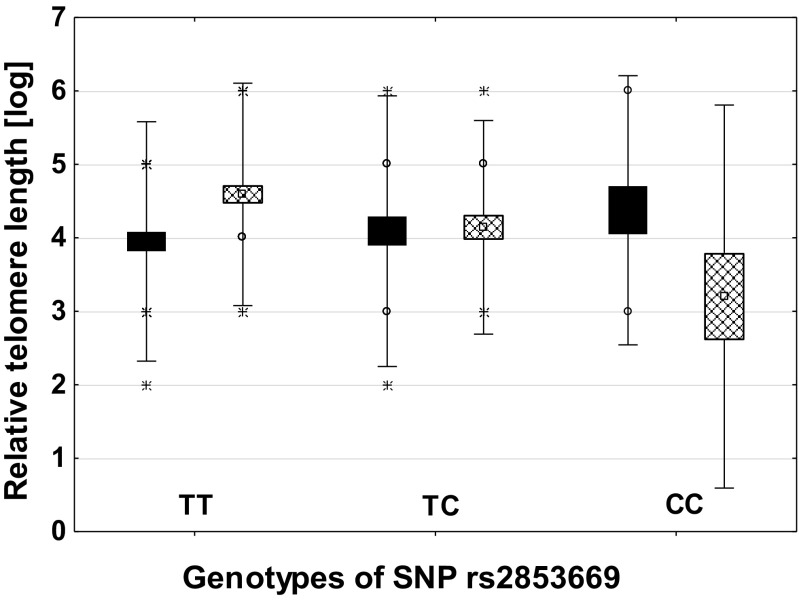
Correlation of relative telomere length (RTL) with the genotypes of the rs2853669 polymorphism in T2DM group and compared to control group. The controls are black boxes, and the T2DM are filled with diagonal check pattern. The values are means + SD (error bars). The outer boxes mark 25–75%. Circles indicate Det. X that indicates Ext.

The rs2853669 genotypes negatively correlated with fasting plasma glucose concentrations in whole study group. However, TC and CC genotypes were accompanied by the lower plasma glucose concentrations (*R*^2^ = 0.03, *p* = 0.03) (Fig. 1). Also, the RTL was lower for TC and CC genotypes of this polymorphism in T2DM study subjects, as calculated from multiple regression analysis (*R*^2^ = 0.11, *p* = 0.005) (Fig. 2). Results presented in Table 2 indicated no elevated risk of T2DM due to the presence of any analysed SNPs. No other statistically significant relations with diabetes-related traits were found (data not shown).

**Table 2:** ORs [95% CI] for associations of the *TERT* SNPs with T2DM risk

Genotype dominant/recessive mode of SNP inheritance	Control subjects (*n* = 70)	T2DM subjects (*n* = 70)	OR [95% CI] *p*
rs2735940 T>C			
TT vs. TC + CC	32 (45.7%) 38 (54.3%)	34 (48.6%) 36 (51.4%)	0.89 [0.46–1.73] *p* = 0.74
TT + TC vs. CC	60 (85.7%) 10 (14.3%)	54 (77.1%) 16 (22.9%)	1.78 [0.74–4.3] *p* = 0.19
rs3215401 insC			
-- vs. -C + CC	40 (57.1%) 30 (42.9%)	41 (58.6%) 29 (41.4%)	0.94 [0.48–1.84] *p* = 0.86
-- + -C vs. CC	68 (97.1%) 2 (2.9%)	67(95.7%) 3 (4.3%)	1.52 [0.25–9.40] *p* = 0.65
rs2853669 T>C			
TT vs. TC + CC	40 (57.1%) 30 (42.9%)	44 (62.9%) 26 (37.1%)	0.79 [0.40–1.55] *p* = 0.49
TT + TC vs. CC	62 (88.6%) 8 (11.4%)	65 (92.9%) 5 (7.1%)	0.59 [0.19–1.92] *p* = 0.37

*OR* odds ratio, *CI* confidence interval

## Discussion

### Relations between RTL and T2DM

Short RTL has been postulated in some population studies as a risk factor for the development of T2DM (Tamura et al. 2016a). Results of the presented work, which was conducted on selected population of elderly obese participants with and without T2DM, did not reveal significant difference in RTL of the two groups. Also, the risk of T2DM reported previously by others was fairly attenuated in postmenopausal women (You et al. 2012). Additionally, no correlations were obtained when some diabetes related traits were analysed. It was hypothesized that telomere attrition would be a marker of insulin resistance (Verhulst et al. 2016); however, in this work, no significant correlation was found between RTL and HOMA-IR. According to other reports, RTL was already reduced in individuals with impaired glucose tolerance (Bethancourt et al. 2017), but high plasma glucose concentration alone did not result in faster telomere shortening (Salpea et al. 2013). Our data do not differ from previously reported findings. One explanation is that T2DM patients usually regularly take glucose-lowering medicines and other formulations; thus, the pathological effect of the disease could be, to some extent, distorted. Additionally, numerous studies have reported that the association between telomere attrition and both types of DM were correlated with the time of the disease duration (Willeit et al. 2014). Considering the fact, that weight gain during adulthood is associated with shortened telomeres, although this dependence is stronger at age under 60 years (Müezzinler et al. 2016), it seems that this phenomenon, but not T2DM itself, is responsible for RTL in the population analysed here.

### Selected *TERT* genotypes in the participants with and without T2DM

In our study, the rs2853669 CC homozygotes had the shortest RTL when compared to other genotypes of this polymorphism. The difference, however, was significant only in T2DM subjects. This SNP is localized in the erythroblast transformation specific - 2 (ETS2) transcription factor consensus binding site. ETS2 is a positive regulator of *TERT* expression and the C allele was shown to reduce telomerase activity (Soerensen et al. 2012). The plasma glucose concentration was lowest in the CC homozygotes presumably due to reduced telomerase activity. It is in correlation with the previous observation that TERT regulates the pentose phosphate pathway; therefore, cells bearing *TERT* mutations promoting telomerase activation exhibited diminished glycogen accumulation (Ahmad et al. 2016).

For the other detected polymorphisms, the genotypes did not differ significantly, although for the SNP rs2735940, the 25% higher promoter activity for genotype TT and longer telomeres were previously reported (Matsubara et al. 2006b). In our studies, this polymorphism showed significant deviation from the Hardy-Weinberg distribution, with higher proportion of homozygotes in T2DM subjects. The observation was correlated with similar results obtained for a larger population (Montesanto et al. 2018). Therefore, we conclude that the presence of rs2735940-C allele could provide additive prognostic value on the presence of T2DM.

Also, the polymorphism rs7712562 showed deviation from H-W disequilibrium in both analysed groups. This observation, together with the statistically important difference of MAF distribution of polymorphism rs2735940 between our control group of old, relatively healthy participants and data presented for Central Europeans by NCBI, USA, gives an impact to further investigation in a larger study group on their influence on longevity. Polymorphisms under study are linked (data not shown); further study on variants combinations can set a new light on the *TERT* promoter functioning. Also, the observed variation in RTL that could not be linked directly neither to T2DM nor to the *TERT* promoter sequence variants indicates that there are also other contributing factors that need more attention.

## General conclusions

Our study has some limitations. One of them is that the absolute Q-PCR results from different laboratories widely differ from each other. Next one is that the telomere length is likely to differ among blood cell types. Finally, the study was conducted on a relatively small sample size, which might have led or partially contributed to the weakness of the observed effects.

On the other hand, our study has a number of strengths. Firstly, the study participants were well selected in terms of morbidity: except for T2DM in the test group, they have had no other diagnosed diseases. In addition, characteristics of both groups were very similar as they differed only by the presence or absence of glucose and lipid disturbances. Secondly, a large fragment of the promoter was sequenced and analysed, including the core promoter region, where no other sequence variations were detected. Further analyses of the promoter/regulatory regions of *TERT* are necessary because the single base changes especially in DNA sequences of transcription factors and/or general transcription factors, recognition/binding sites might affect *TERT* transcription initiation and its rate.

## Electronic supplementary material

Table S1 Distribution of the *TERT* alleles in healthy obese controls and obese diabetic (T2DM) elderly subjects. (DOCX 23 kb)
