# Supplementary material for: Possible association of the TERT promoter polymorphisms rs2735940, rs7712562 and rs2853669 with diabetes mellitus in obese elderly Polish population: results from the national PolSenior study
Source: J Appl Genet. 2018 Jun 25;59(3):291–9. doi: 10.1007/s13353-018-0450-9 (PMC6060992; doi:10.1007/s13353-018-0450-9)
Supplement: Supplementary file 1 — Distribution of the TERT alleles in healthy obese controls and obese diabetic (T2DM) elderly subjects. (DOCX 23 kb) [file 13353_2018_450_MOESM1_ESM.docx]

**Table S1. Distribution of the *TERT* alleles in healthy obese controls and obese diabetic (T2DM) elderly subjects** (Montesanto et al. 2018)**.**

| Genotype  MAF | Controls  (n=70) | T2DM Subjects  (n=70) | All participants  (n=140) |
| --- | --- | --- | --- |
| **rs2735940** | | | |
| TT | 32 (45.7%) | 34 (48.6%) | 66 (47.1%) |
| TC | 28 (40%) | 20 (28.6%) | 48 (34.3%) |
| CC | 10 (14.3%) | 16 (22.8%) | 26 (18.6%) |
| MAF | 0.34 | 0.37* | 0.36 |
| MAF NCBI | 0.49 |  |  |
| MAF (1) | 0.415 |  |  |
| **Rs3215401** | | | |
| -- | 40 (57.1%) | 41 (58.6%) | 81 (57.9%) |
| -C | 28 (40%) | 26 (37.1%) | 54 (38.6%) |
| CC | 2 (2.9%) | 3 (4.3%) | 5 (3.6%) |
| MAF | 0.23 | 0.23 | 0.23 |
| MAF NCBI | 0.29 |  |  |
| **Rs7712562** | | | |
| TT | 13 (18.6%) | 11 (15.7%) | 24 (17.1%) |
| TC | 15 (21.4%) | 8 (11.4%) | 23 (16.4%) |
| CC | 42 (60%) | 51 (72.9%) | 93 (66.4%) |
| MAF | 0.29** | 0.21** | 0.25 |
| MAF NCBI | 0.15 |  |  |
| **rs33958877** | | | |
| CC | 69 (98.6%) | 67 (95.7%) | 136 (97%) |
| CA | 1 (1.4%) | 2 (2.9%) | 3 (2.2%) |
| AA | 0 (0%) | 1 (1.4%) | 1 (0.7%) |
| MAF | 0.01 | 0.03** | 0.015 |
| MAF NCBI | 0.04 |  |  |
| **rs35161420** | | | |
| GG | 69 (98.6%) | 67 (95.7%) |  |
| GC | 1 (1.4%) | 3 (4.3%) |  |
| CC | 0 (0%) | 0 (0%) |  |
| MAF | 0.01 | 0.03 |  |
| MAF NCBI | 0.04 |  |  |
| **rs35226131** | | | |
| GG | 68 (97.1%) | 67 (95.7%) |  |
| GA | 2 (2.9%) | 3 (4.3%) |  |
| AA | 0 (0%) | 0 (0%) |  |
| MAF | 0.01 | 0.04 |  |
| MAF NCBI | 0.04 |  |  |
| **rs2853669** | | | |
| TT | 40 (57.1%) | 44 (62.9%) | 84 (60.0%) |
| TC | 22 (31.4%) | 21 (30%) | 43 (30.7%) |
| CC | 8 (11.4%) | 5 (7.1%) | 13 (9.3%) |
| MAF | 0.27 | 0.22 | 0.25 |
| MAF NCBI | 0.3 |  |  |

MAF: Minor Allele Frequency. NCBI: data from the database of the National Centre for Biotechnology Information, USA, for Caucasians from Central Europe. The probability calculated for deviations from Hardy-Weinberg equilibrium model: *: p<0.01; **: p<0.001.

Underlined case: SNPs, which underwent subsequent statistical analyses

(1) Montesanto A, Bonfigli AR, Crocco P, Garagnani P, De Luca M, Boemi M, et al. Genes associated with Type 2 Diabetes and vascular complications. Aging (Albany NY) [Internet]. 2018 [cited 2018 Apr 12];10(2). Available from: www.aging‐us.com
